# Development and Practical Application of Glucose Biosensor Based on Dendritic Gold Nanostructures Modified by Conducting Polymers

**DOI:** 10.3390/bios12080641

**Published:** 2022-08-14

**Authors:** Natalija German, Anton Popov, Arunas Ramanavicius, Almira Ramanaviciene

**Affiliations:** 1 Department of Immunology, State Research Institute Centre for Innovative Medicine, LT-08406 Vilnius, Lithuania; 2 NanoTechnas—Center of Nanotechnology and Materials Science, Institute of Chemistry, Faculty of Chemistry and Geosciences, Vilnius University, LT-03225 Vilnius, Lithuania; 3 Department of Physical Chemistry, Faculty of Chemistry and Geosciences, Vilnius University, LT-03225 Vilnius, Lithuania

**Keywords:** dendritic gold nanostructures, electrochemical methods, glucose oxidase, polyaniline, polypyrrole, biomedical samples and beverages

## Abstract

In this study, graphite rod (GR) electrodes were electrochemically modified by dendritic gold nanostructures (DGNs) followed by immobilization of glucose oxidase (GOx) in the presence of mediator phenazine methosulfate (PMS). Modified with polyaniline (PANI) or polypyrrole (Ppy), GOx/DGNs/GR electrodes were used in glucose biosensor design. Different electrochemical methods were applied for the registration of glucose concentration, and constant potential amperometry (CPA) was chosen as the best one. PANI and Ppy layers synthesized enzymatically on the GOx/DGNs/GR electrodes extended the linear glucose determination range, the width of which depended on the duration of PANI- and Ppy-layers formation. Enzymatically formed polypyrrole was determined as the most suitable polymer for the modification and formation of the glucose biosensor instead of polyaniline, because it was 1.35 times more sensitive and had a 2.57 times lower limit of detection (LOD). The developed glucose biosensor based on the Ppy/GOx/DGNs/GR electrode was characterized by appropriate sensitivity (59.4 μA mM^−1^ cm^−2^), low LOD (0.070 mmol L^−1^), wide linear glucose determination range (up to 19.9 mmol L^−1^), good repeatability (8.01%), and appropriate storage stability (33 days). The performance of the developed glucose biosensor was tested in biological samples and beverages.

## 1. Introduction

The emergence of nanotechnology has opened up new horizons for the development of electrochemical [1,2,3,4,5] and optical [6] biosensors. Carbon-based nanomaterials are promising for the construction of biosensors and other electrochemical sensors, in catalysis, batteries, fuel cells, and photovoltaic devices [7,8,9]. Characterized by fast electron-transfer kinetics and excellent sensing performance, electrochemical biosensors are widely applied for food control [10,11,12] and disease diagnosis [3,5]. Diabetic people constitute about 5% of population; therefore, sensitive, selective, and easy-to-use glucose biosensors are very important for the diagnosis and control of diabetes [3,13,14]. Electrochemical biosensors based on chronoamperometry (ChA) and constant potential amperometry (CPA) [15,16] and differential pulse voltammetry (DPV) [11] are usually applied for glucose determination.

Enzymatic electrochemical biosensors are characterized by high sensitivity and selectivity due to enzyme specificity and effective electron transfer between covalently linked glucose oxidase (GOx) or immobilized quinoprotein glucose dehydrogenase (GDH) and the electrode surface [17,18,19,20]. Oxidative processes in glucose biosensors involve charge transfer processes between glucose, glucose oxidase, redox mediators, molecular oxygen, nanomaterials, and formed hydrogen peroxide [21,22]. GOx is the dimeric enzyme most commonly used in glucose determination [3,20]. Each monomer of GOx contains a strongly bounded and deeply buried flavin adenine dinucleotide (FAD) cofactor [3,7]. GOx-FADH_2_ formed during enzymatic oxidation of glucose is reoxidized back into oxidized form with O_2_ or the artificial redox mediator (replace natural electron acceptor) used in the 2nd generation of glucose biosensors. Redox mediators such as potassium ferrocianyde (K_4_[Fe(CN)_6_]) [9,12,19], phenazine methosulfate (PMS) [23,24], and many others are used to facilitate charge transfer between the redox-active center of the enzyme and the electrode surface [10].

In recent years, the number of researches on the application of nanomaterials, such as nanorods [20,25], nanoparticles [6,26,27,28], nanostructures [29,30,31,32], nanosheets [33,34], nanotubes, and nanowires [35], has increased. Some notable properties of these materials, such as high surface area and the ability to facilitate charge transfer between the redox center of the enzyme and the electrode, are exploited in the design of biosensors to improve sensitivity and selectivity and reduce response time [15]. Dendritic gold nanostructures (DGNs) are novel nanomaterials that show great promise in various biomedical applications. The complex of gold dendrimer-nanoparticles and platinum (Pt) nanoparticles can enhance the electrocatalytic ability of the modified surface [16]. The most popular method of synthesis of DGNs is their electrochemical deposition from aqueous solutions of hydrogen tetrachloroaurate (III) (HAuCl_4_) [15,30,31,32]. During the formation of DGNs, initially, AuCl_4_^−^ ions present in the solution are slowly reduced to Au^0^ [4,32], and their concentration increases rapidly in the solution until the nucleation process begins [8]. Then nucleated Au^0^ forms clusters following the formation of dendrites [32,36].

Various conducting polymers such as polyaniline (PANI) [37,38] and polypyrrole (Ppy) [24,39] are widely used in electrocatalysis and for the immobilization of biological molecules. Usually, conducting polymers are formed by chemical and electrochemical polymerization of corresponding monomers [29,38]. Enzymatic formation of conducting polymers, which is based on the initiation of an enzymatic polymerization reaction by formed H_2_O_2_, is a rather simple one-step process [27,40], which is suitable to tune the analytical characteristics of amperometric glucose biosensors. During the polymerization reaction, the immobilized GOx is entrapped within the formed polymer layer. Additionally, it was determined that the polymerization rate is almost similar in a broad pH range for Ppy and PANI (from pH 2.0–3.0 to 9.0) [41,42]. Other advantages of enzymatic polymerization are the control of reaction kinetics, simplicity, and the good activity of the enzyme in aqueous solutions [40]. The enzymatic polymerization rate is affected by the temperature, concentration of reaction compounds, and additives [43]. Ppy layers can be used to reduce interference by electrochemically active compounds such as ascorbic acid (AA) and uric acid (UA) [2].

The main aim of the investigations presented here was to evaluate the efficiency of PANI and Ppy layers formed by enzymatic polymerization on the surface of the graphite rod (GR) electrode initially premodified by electrochemically synthesized DGNs and drop-casted GOx. Analytical characteristics of biosensors based on PANI/GOx/DGNs/GR and Ppy/GOx/DGNs/GR electrodes were evaluated and compared. The applicability of improved glucose biosensors based on the Ppy/GOx/DGNs/GR electrodes was assessed in various real samples. The novelty of this manuscript is based on the advantages of the combination of dendritic gold nanostructures with a polymer layer to improve storage stability and resistance to the interfering species and to extend the linear glucose determination range in the real samples.

## 2. Materials and Methods

### 2.1. Materials

201 units mg^−1^ protein of GOx (EC 1.1.3.4, type VII, from *Aspergillus niger*) was purchased from Fluka (Buchs, Switzerland), sugars (D-(+)-glucose, D(+)-saccharose, D(+)-xylose, D(+)-galactose, D(+)-mannose, D(−)-fructose) and tannic acid—from Carl Roth GmbH + Co.KG (Karlsruhe, Germany), potassium nitrate (KNO_3_)—from Acros Organic (New Jersey, NJ, USA). To prepare 0.05 mol L^−1^ sodium acetate (SA) buffer with 0.1 mol L^−1^ potassium chloride (KCl), sodium acetate trihydrate (CH_3_COONa·3H_2_O) from Reanal (Budapest, Hungary) and KCl from Lachema (Neratovice, Czech Republic) were mixed together. Sodium dihydrogen phosphate dihydrate (NaH_2_PO_4_ 2H_2_O), disodium hydrogen phosphate dodecahydrate (Na_2_HPO_4_·12H_2_O) from Lachema (Neratovice, Czech Republic), and KCl were mixed to prepare 0.05 mol L^−1^ sodium phosphate (SP) buffer with 0.1 mol L^−1^ KCl. The sodium acetate and sodium phosphate (S-AP) buffer was prepared from 0.05 mol L^−1^ CH_3_COONa·3H_2_O, 0.05 mol L^−1^ NaH_2_PO_4_·2H_2_O, and Na_2_HPO_4_·12H_2_O solutions mixed with 0.1 mol L^−1^ KCl. Aniline and pyrrole were purchased from Merck KGaA (Darmstadt, Germany) and Acros Organics (New Jersey, NJ, USA), respectively. Hydrogen tetrachloroaurate(III) trihydrate and GR (3 mm of the diameter) were obtained from Sigma-Aldrich (Saint Louis, MO, USA); PMS, AA, and UA—from AppliChem GmbH (Darmstadt, Germany); 25% glutaraldehyde—from Fluka Chemie GmbH (Buchs, Switzerland).

### 2.2. Pretreatment, Immobilization, and Modification of the Working Electrode

The electrochemical synthesis of long, thin, and branch DGNs on the polished surface of the GR electrode was carried out according to previously described methodology [44]. Shortly, synthesis was performed using the CPA method (400 s, applied potential (*E*_app_) was −0.4 V) from solution containing 6 mmol L^−1^ HAuCl_4_ and 0.1 mol L^−1^ KNO_3_. Then, 3 µL of 25 mg mL^−1^ GOx solution was deposited on the DGNs/GR electrode. After solvent evaporation, the GOx/DGNs/GR electrode was stored for 15 min over 25% glutaraldehyde solution at +20 ± 2 °C.

Enzymatic polymerization of aniline or pyrrole on electrodes modified with DGNs and GOx was carried out in a solution of 0.2 mol L^−1^ of the corresponding monomer and 0.05 mol L^−1^ glucose in 0.05 mol L^−1^ SA buffer (pH 6.0). Deposition was fulfilled in the dark at room temperature for different times of polymerization (22, 44 and 68 h). Electrodes were stored between measurements over the buffer solution at +4 °C. Detailed information about the principle of the enzymatic polymerization was published in our previous articles [45,46,47]. Shortly, the GOx catalyzes the oxidation of β-D-glucose to D-glucono-δ-lactone (in water it is hydrolyzed to β-D-gluconic acid and pH is reduced towards the acidic pH) and H_2_O_2_ in the presence of dissolved oxygen. Formed H_2_O_2_ is able to generate free radical cations of pyrrole or aniline and initiate the polymerization reaction following the oligomers’ and polymeric structures’ formation.

### 2.3. Electrochemical Measurements

Electrochemical measurements during the registration of the current response towards different glucose concentrations were performed using a computerized potentiostat/galvanostat PGSTAT 30/Autolab (EcoChemie, Netherlands). A three-electrodes system was used in all electrochemical measurements: a working electrode—GOx/DGNs/GR, PANI/GOx/DGNs/GR or Ppy/GOx/DGNs/GR; an auxiliary electrode—2 cm^2^ platinum spiral; and a reference electrode—Ag/AgCl_(3 mol L_^−1^_KCl)_ from Metrhom (Herisau, Switzerland).

To select the most optimal buffer among several available, the CPA was used, and the biosensor response against different glucose concentrations in stirred (1200 rpm) 0.05 mol L^−1^ SA, SP, or S-AP buffers (pH 6.0) with 0.1 mol L^−1^ KCl and 6 mmol L^−1^ of PMS (oxidized form) at +0.3 V vs. Ag/AgCl_(3 mol L_^−1^_KCl)_ was evaluated.

The selection of the most efficient electrochemical method for glucose current response registration was performed in 0.05 mol L^−1^ SA buffer in the presence of 6 mmol L^−1^ PMS using CPA, DPV, and square wave-voltammetry (SWV). For CPA-based experiments, *E*_app_ = +0.3 V was applied; for DPV—the potential (*E*_sc_) was scanned from –0.6 to +0.8 V, the step potential (*E*_s_) of 0.025 V, the amplitude (Δ*E*) of 0.025 V, the scan speed (*v*) of 0.05 V s^−1^; for SWV—*E*_sc_ from −0.6 to +0.8 V, *E*_s_ = 0.050 V, Δ*E* = 0.025 V, *v* = 0.05 V s^−1^; frequency of 10 Hz. The cyclic voltammetry (CV) with the potential scan from −0.6 to +0.8 V, the step potential of 0.025 V and the potential scan rate of 0.10 V s^−1^ was applied to evaluate the reversibility of the redox reaction in an unstirred solution of SA buffer (pH 6.0).

Electrochemical measurements were performed in the buffer solution with a glucose concentration range from 0.10 to 97.0 mmol L^−1^. The oxidation of reduced phenazine methosulfate form (PMSH_2_) and/or H_2_O_2_ produced by the enzymatic reaction were measured at +0.3 V vs. Ag/AgCl_(3 mol L_^−1^_KCl)_ to evaluate the glucose concentration [23]. During stability experiments, the fabricated electrodes between measurements were stored at +4 °C in a closed vessel hanging over SA buffer solution.

**Figure 1:**
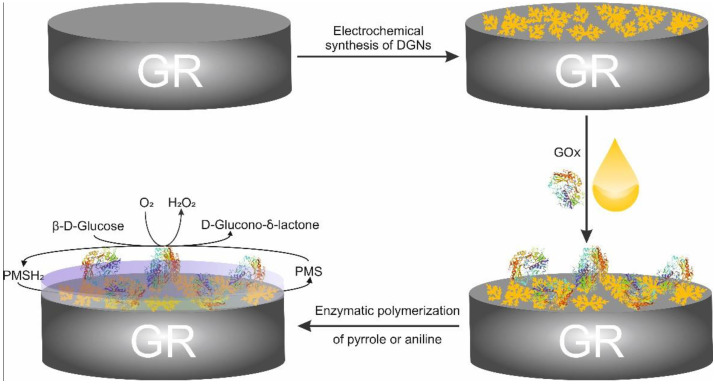
Schematic representation of GR electrode modification with dendritic gold nanostructures with the following immobilization of GOx and the enzymatic formation of the polymer (PANI or Ppy) layer for the electrochemical glucose determination.

The principle of DGNs electrochemical formation, enzymatic polymerization on GR electrode, and electrochemical investigation are represented in Figure 1. PMS as a redox mediator reoxidizes the GOx-FADH_2_, and electrons from PMSH_2_ are transferred towards the positively charged GR electrode through DGNs.

### 2.4. The Assessment of Glucose Biosensor Based on a Ppy/GOx/DGNs/GR Electrode in Real Samples

Real samples (human serum, saliva, wine, milk, juice) were diluted in 0.05 mol L^−1^ SA (pH 6.0) and centrifuged for 8 min by an IEC CL31R Multispeed centrifuge (14.6 × 10^3^× *g*, France) (approval of the ethics committee is not required for the research with a sample from a single person and for one-time use). All measurements were performed with a biosensor based on the Ppy/GOx/DGNs/GR electrode. Electrochemical investigations were performed in 10-times-diluted samples of human serum and saliva, wine and 100-times-diluted samples of milk, and juices. The selectivity of fabricated electrodes in diluted real samples was evaluated by monitoring the analytical signal; then, 10.0 mmol L^−1^ of glucose and 1 mmol L^−1^ of saccharose, xylose, galactose, mannose, and fructose were sequentially added to the solutions. Moreover, evaluation of the addition of AA and UA as electroactive species to various real samples was performed. In such experiments, the generated current was monitored after the addition of 10.0 mmol L^−1^ glucose in the presence and absence of AA and UA at concentrations 0.01 or 0.05 mmol L^−1^ and 0.01 or 0.1 mmol L^−1^, respectively.

### 2.5. Calculations

At least three measurements were performed for each point on the calibration curves. Software SigmaPlot (version 12.5) was applied for the calculation of the limit of detection (LOD) and the parameters of Michaelis-Menten kinetics. The LOD value was calculated as the lowest concentration of analyte, the addition of which generates a current response greater than the background value plus 3 σ. Error bars are presented as the standard deviation of independent measurements of 3–5 electrodes.

## 3. Results and Discussion

### 3.1. The Elaboration of Optimal Conditions for the Determination of Glucose

**Figure 2:**
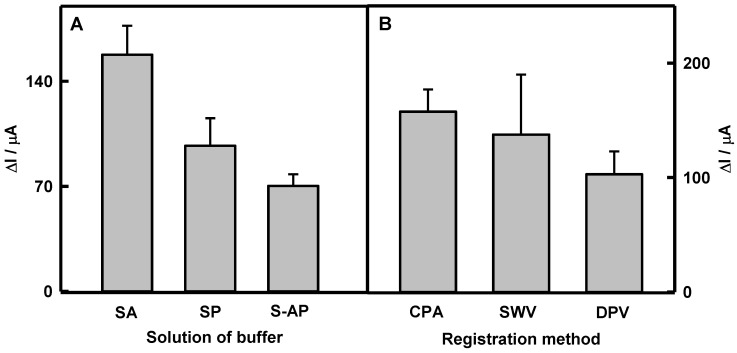
Diagrams of the current response registered (**A**) using the GOx/DGNs/GR electrode in different buffer solutions (pH 6.0) by the CPA method and (**B**) applying different electrochemical methods in SA buffer (pH 6.0). All measurements were performed in the presence of 6.0 mmol L^−1^ PMS and 27 mmol L^−1^ glucose; all buffer solutions contained 0.1 mol L^−1^ KCl.

First of all, the effect of the nature of the buffer solution on the response of a glucose biosensor based on GOx/DGNs/GR was studied. Three different buffer solutions (SA, SP, and S-AP) were used, and the response of the biosensor was monitored using the CPA method (Figure 2A). It was found that the highest analytical signal could be registered using SA buffer solution. The current response to glucose in SA buffer (158 ± 19.3 μA) was 1.63 and 2.25 times higher than that in SP (97.0 ± 18.3 μA) and S-AP (70.3 ± 7.7 μA) buffers, respectively. The ratio “current response/noise” of S-AP buffer (9.12) is higher by 1.1 and 1.7 times than that of SA (8.19) or SP (5.30) buffers. However, the difference of this ratio between SA and S-AP buffers is insignificant; therefore, more attention has been paid to the magnitude of the current response, which was the most relevant for polymer-modified electrodes. For this reason, 0.05 mol L^−1^ SA buffer (pH 6.0) with 0.1 mol L^−1^ KCl was chosen as optimal.

The sensitivity of biosensors depends on the applied electrochemical method for registration of the analytical signal. To select the most efficient electrochemical method, investigations were carried out using three different electrochemical methods—CPA, SWV, and DPV, whose original data plots are presented in Figure S1. As can be seen from the presented results, three peaks appeared at −0.260, −0.097, and +0.410 in SWV (Figure S1B) and at −0.200, −0.040, and +0.469 V in DPV (Figure S1C) voltammograms in the presence of 6 mmol L^−1^ PMS without glucose (blue lines). At the same potential values, the peak positions are observed after the addition of 27 mmol L^−1^ glucose. Only the second peak for both cases at the potential of +0.071 V increases significantly, which means that the electrochemical process takes place on the surface of electrode in the presence of analyte. The other peaks are related to PMS oxidation/reduction on the electrocatalytically active surface of GOx/DGNs/GR electrode. Biosensor responses are evaluated as the differences between peaks at +0.071 V in the absence and presence of glucose. Obtained results are compared in Figure 2B. The current response registered by the CPA method was determined to be 1.14 and 1.53 times higher than that registered by the SWV and DPV methods. In addition, despite a similar analytical signal using CPA and SWV methods, the errors were significantly lower in case of CPA. Therefore, the CPA method was selected for further investigations. Additionally, charge transfer ability on modified GOx/DGNs/GR electrode was investigated by cyclic voltammetry (Figure S2). It is seen that without the redox mediator, the GR electrode is characterized by narrow and smooth cyclic voltammogram, and no electrochemical reaction occurs on the surface of electrode (Figure S2, black dashed line). After the addition of PMS to the GOx/DGNs/GR electrode, the form of the cyclic voltammogram was expanded and was characterized by the reversibility of redox reaction. The anodic and cathodic peaks monitored using the GOx/DGNs/GR electrode in the presence of PMS and without glucose (Figure S2, blue line) were noticeable at *E*_pa_ = +0.005 and *E*_pc_ = −0.225 V vs. Ag/AgCl_(3 mol L_^−1^_KCl)_. The reversibility was evaluated by the calculation of peak separation (Δ*E*_p_) using the formula Δ*E*_p_ *= E*_pa_ − *E*_pc_, and it was calculated as 0.23 V vs. Ag/AgCl_(3 mol L_^−1^_KCl)_, which means that the redox process is diffusion-controlled and reversible with fast electron transfer [12]. The addition of 27 mmol L^−1^ glucose increased the current response of the biosensor based on the GOx/DGNs/GR electrode (Figure S2, black line). According to the presented cyclic voltammograms, it is most reasonable to apply +0.3 V potential for the registration of current response by the CPA method.

### 3.2. The Influence of PANI and Ppy Enzymatically Formed Layers on Current Responses of Biosensors Based on the PANI/GOx/DGNs/GR or Ppy/GOx/DGNs/GR Electrodes

The most important advantage of enzymatic polymerization is the improvement of the analytic characteristics of the biosensors by the deposited conducting polymer layer, which can be formed over the other layers of the biosensing structure. One of the most important parameters of polymerization reaction is the duration of this process, because it affects the thickness and density of the formed polymer layer. The diameter of Ppy nanoparticles determined by an atomic force microscope was 25 ± 10 nm and 80 ± 20 nm after 24 and 96 h of enzymatic polymerization, respectively [45]. Thus, the thickness of the polymer’s layers after 68 h of polymerization has to be less than 100 nm.

**Figure 3:**
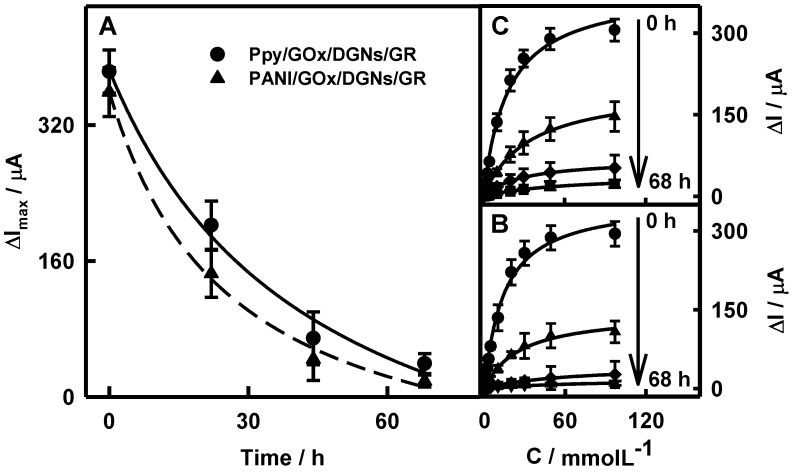
The influence of enzymatic polymerization duration on the current response (**A**) and calibration plots registered using PANI/GOx/DGNs/GR (**B**) or Ppy/GOx/DGNs/GR (**C**) electrodes.

The influence of enzymatically formed PANI and Ppy layers on the current response and hyperbolic dependence of CPA signals towards glucose in the concentration range from 0.10 to 97.0 mmol L^−1^ were also determined for the PANI/GOx/DGNs/GR and Ppy/GOx/DGNs/GR electrodes (Figure 3). The formation of the Ppy or PANI layer on the surface of the GOx/DGNs/GR electrode was carried out for 22, 44, and 68 h. This process is very sensitive to many parameters: DGNs were individually formed on each electrode, the enzyme solution was also individually deposited on the DGNs/GR, and the coverage of the surface area and orientation of enzyme was random. The error bars were calculated from results obtained from a minimum of 3–5 different electrodes. It can be seen that Michaelis-Menten kinetics well describe the dependence of the analytical signal on glucose concentration. (Figure 3B,C). The maximal current (Δ*I*_max_) was used as parameter *a* of the hyperbolic function when the apparent Michaelis constant (*K*_M(apparent)_) was applied as parameter *b*.

The decrease of the analytical signal was observed while the duration of the polymerization increased, regardless of which polymer was formed. As is seen from Figure 3A, after 22, 44, and 68 h of enzymatic polymerization in aniline or pyrrole solutions, Δ*I*_max_ decreased by 2.48, 8.12, and 19.3 and 1.90, 5.55, and 9.75 times, respectively, if compared with the Δ*I*_max_ registered by the GOx/DGNs/GR electrode. After 22 h of aniline polymerization, the current response was 1.3 times lower than using pyrrole for polymeric layer formation. The main reason for those differences could be the faster formation of the PANI layer, which causes an increase in the thickness of the polymer layer on the surface.

Thus, *K*_M(apparent)_ equal to 13.8 mmol L^−1^ for the unmodified by polymer electrode was determined. Increasing the polymerization duration from 22 to 68 h, the value of *K*_M(apparent)_ increased from 26.2 to 75.2 and from 29.6 to 61.8 mmol L^−1^ for the PANI/GOx/DGNs/GR and Ppy/GOx/DGNs/GR electrodes, respectively. Such an effect can be associated with the diffusion limitation of glucose towards GOx molecules through the polymer layer [23], and could be the evidence of enzyme entrapment in the polymer layer. In addition, there was a decrease in the Δ*I*_max_ of biosensors caused by the hindered diffusion of PMS and glucose. Moreover, the increased *K*_M(apparent)_ correlates with the extension of the linear glucose determination range. This is considered as one of main advantages, which is provided by the formation of the polymer layer on the surface of the GOx/DGNs/GR electrode.

### 3.3. Analytical Characteristics of Biosensors Based on the PANI/GOx/DGNs/GR and Ppy/GOx/DGNs/GR Electrodes

**Figure 4:**
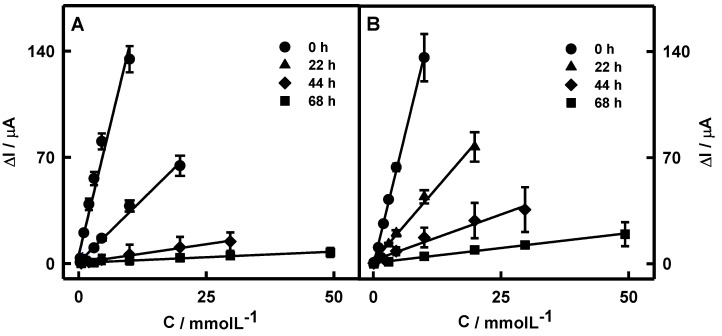
The linear glucose determination ranges of biosensors based on the PANI/GOx/DGNs/GR (**A**) and Ppy/GOx/DGNs/GR (**B**) electrodes when polymerization was performed for 0, 22, 44, and 68 h.

The influence of PANI or Ppy layers on the linear glucose determination range was presented in Figure 4A,B, respectively. It was observed that the linear glucose determination ranges for both PANI/GOx/DGNs/GR and Ppy/GOx/DGNs/GR electrodes increased along with the extended duration of PANI and Ppy layer formation, which indicates the suitability of the designed electrodes for glucose biosensing. The linear glucose determination ranges for polymer-modified PANI/GOx/DGNs/GR and Ppy/GOx/DGNs/GR electrodes for 22-, 44-, and 68 h-lasting enzymatic polymerization were extended up to 19.9, 29.7, and 49.3 mmol L^−1^, respectively, and was 2.00, 2.98, and 4.95 times wider than that registered by the GOx/DGNs/GR electrode (9.96 mmol L^−1^). As an example, the linear glucose determination ranges for four Ppy/GOx/DGNs/GR electrodes after 22 h of polymerization are presented in Figure S3. It is seen that the mean of the current responses well correlates with results obtained by the four electrodes and might be used for the further evaluation. The linear glucose determination range after 22 h of enzymatic polymer synthesis was far beyond the physiological level of the glucose in the human serum sample (3–8 mmol L^−1^) [31]. Therefore, such a biosensor can be used for the determination of glucose in real samples. 

The sensitivity of the designed biosensors based on the PANI/GOx/DGNs/GR and Ppy/GOx/DGNs/GR electrodes after 22, 44, and 68 h of the polymer’s synthesis was determined as 43.9, 7.46, and 3.05 and 59.4, 24.7, and 5.83 μA mM^−1^ cm^−2^, respectively. Biosensors based on the PANI/GOx/DGNs/GR or Ppy/GOx/DGNs/GR electrodes after 22, 44, and 68 h of the polymer’s formation were 3.58, 21.0, and 51.5 and 2.64, 6.36, and 26.9 times less sensitive than that based on the GOx/DGNs/GR electrode (157 μA mM^−1^ cm^−2^). Obviously, the sensitivity of biosensors based on the PANI/GOx/DGNs/GR or Ppy/GOx/DGNs/GR electrodes decreased more significantly if the formation of the PANI and Ppy layers was applied for longer periods of time. The 22 h period was found to be the most optimal for PANI or Ppy layer formation, maintaining rather high sensitivity and wide linear glucose determination range.

The linear ranges of designed glucose biosensors based on the PANI/GOx/DGNs/GR or Ppy/GOx/DGNs/GR electrodes after 22 h of polymerization were characterized by *R^2^* 0.9818 and 0.9803, respectively. The biosensor based on the Ppy/GOx/DGNs/GR electrode was 1.35 times more sensitive than that based on the PANI/GOx/DGNs/GR electrode. It should be noted that the PANI/GOx/DGNs/GR electrode was characterized by higher sensitivity if compared with electrodes based on GOx, gold compounds, and polymers which were reported in another researches [23,25]. The sensitivity of the glucose biosensor based on electrochemically deposited 13 nm gold nanoparticles (AuNPs), immobilized GOx, and enzymatically formed Ppy layer on the GR electrode was 21.7 μA mM^−1^ cm^−2^ [23]. The sensitivity of the glucose biosensor based on the Pt electrode directly modified by the gold nanorod composite, GOx, and electropolymerized PANI was 13.8 μA mM^−1^ cm^−2^ [25].

The repeatability of biosensors fabricated using PANI and Ppy at 1.00 mmol L^−1^ of glucose was 8.68 and 8.01%, respectively. Moreover, a relatively fast biosensor response was monitored for both biosensors. After the addition of 10.0 mmol L^−1^ glucose, 95% of the analytical signal was achieved within the initial 5 s. The linear dependencies for PANI/GOx/DGNs/GR and Ppy/GOx/DGNs/GR electrodes in the range of glucose concentration from 0.10 until 1.00 mmol L^−1^ (Figure S4) are characterized by rather small error bars and were used to estimate the limit of detection. The biosensors fabricated using Ppy and PANI exhibited LOD values of 0.070 and 0.18 mmol L^−1^, respectively. In the further research, a Ppy-modified electrode was used, due to higher sensitivity and lower LOD value in comparison with the PANI/GOx/DGNs/GR electrode. The properties of designed biosensors were compared with other recently developed glucose biosensors based on gold derivatives (Table 1). As can be seen, the developed glucose biosensor based on the Ppy/GOx/DGNs/GR electrode exhibits good performance. For instance, the LOD value of such an electrode was 2.86 times lower compared to the LOD calculated for the GR electrode, which was modified using 13 nm AuNPs and the Ppy layer [23].

### 3.4. The Stability of Glucose Biosensor Based on the Ppy/GOx/DGNs/GR Electrode

**Figure 5:**
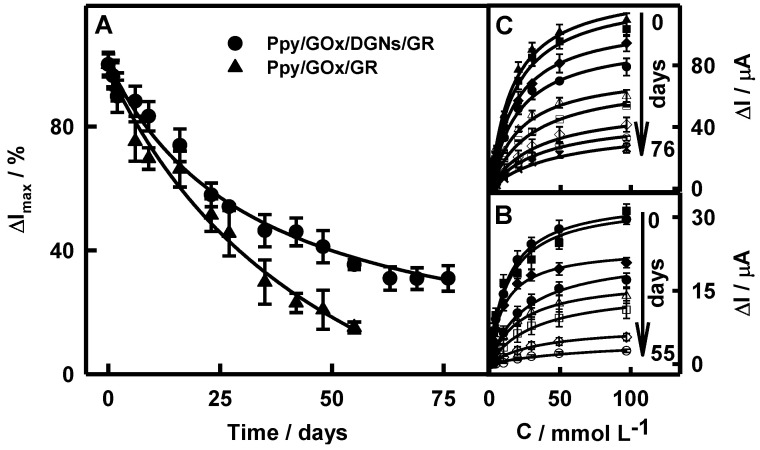
The changes of current responses over time (**A**) and calibration plots of biosensors based on the Ppy/GOx/GR (**B**) or Ppy/GOx/DGNs/GR (**C**) electrodes after 22 h of polymerization.

The next stage of the presented study was the investigation of the stability of the glucose biosensor based on the Ppy/GOx/DGNs/GR electrode and the comparison with results registered using the Ppy/GOx/GR electrode. Positively charged Ppy, which is formed during enzymatic synthesis, is electrostatically attracted to the negatively charged surface of GOx [39]. Moreover, strong interaction between chains of Ppy enables the deposition and aggregation of newly composed Ppy chains [49]. The layer of Ppy significantly hindered the diffusion of glucose, reaction products, and PMS. The stability assessment of the Ppy/GOx/GR and Ppy/GOx/DGNs/GR electrodes was performed, comparing current responses towards the same glucose concentrations in time (Figure 5A) and calibration plots (Figure 5B and 5C, respectively). As seen in Figure 5B,C, the hyperbolic dependences of current responses on analyte concentration were in an agreement with Michaelis-Menten kinetics.

The current responses of the Ppy/GOx/GR and Ppy/GOx/DGNs/GR electrodes toward glucose gradually decreased during the first 16 days to 66.2 and 73.9%, respectively (Figure 5A). From 23 to 35 days, current responses decreased from 51.4 to 29.7 and from 57.9 to 46.3%, respectively. The *τ*_1/2_ for biosensors based on the Ppy/GOx/GR and Ppy/GOx/DGNs/GR electrodes were 22 and 33 days, respectively. The glucose biosensor fabricated using Ppy was 1.50 times more stable than electrode without Ppy (*τ*_1/2_ = 22 days) [44], and 1.50 and 1.94 times more stable than the GOx/PANI-AuNPs_(6nm)_-GOx/GR (*τ*_1/2_ = 22 days) or GOx/Ppy-AuNPs_(6nm)_-GOx/GR (*τ*_1/2_ = 17 days) electrodes [48]. Moreover, use of Ppy made it possible to achieve 3.37 and 4.71 times higher stability in comparison with biosensors based on the Ppy/GOx/AuNPs(_el depos.)_/GR electrode (*τ*_1/2_ = 9.8 days) [23] and based on the GOx/PANI/Au nanorods/Pt electrode (*τ*_1/2_ = 7.0 days) [25], respectively. The registered current response decay might be based on the decreased activity of the enzyme during the storage and repeated measurements of the Ppy/GOx/DGNs/GR electrode, as was reported in previous researches [46,50].

### 3.5. The Influence of Interfering Species on the Current Response of Designed Biosensor and the Accuracy of Glucose Assessment in Real Samples

**Figure 6:**
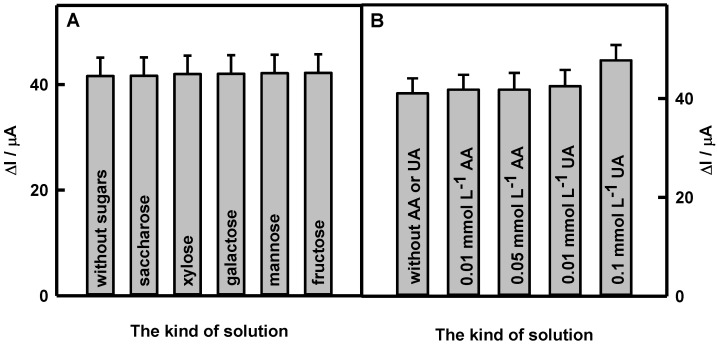
The influence of 1.0 mmol L^−1^ sugars (**A**) and electroactive species (**B**) on the current responses of the glucose biosensor based on the Ppy/GOx/DGNs/GR electrode in the presence of 10.0 mmol L^−1^ glucose. Enzymatic polymerization was performed for 22 h; current responses were registered in the presence of 6 mmol L^−1^ PMS at +0.3 V vs. Ag/AgCl/KCl_(3 mol L_^−1^_)_.

Glucose and fructose are the most relevant sugars for detection in real samples [10]. Saccharose, xylose, galactose, mannose, and fructose are stereoisomers of glucose with the same molecular formula and sequence of bonded atoms. Interfering materials such as ascorbic and uric acids are electrochemically active and are able to affect the electrochemical current response of the glucose biosensor [10,17,25]. The main contribution of the carboxylate group presented in polymeric structures is the exclusion of the matrix effects [25]. The selectivity tests of the glucose biosensor to saccharose, xylose, galactose, mannose, and fructose, as well as to electrochemically active AA and UA, were conducted and presented in this paper. As an example, the influence of interfering species on the current response of the Ppy/GOx/DGNs/GR electrode was tested in a 10-times-diluted sample of human serum containing 10.0 mmol L^−1^ glucose after the addition of 1.0 mmol L^−1^ saccharose, xylose, galactose, mannose, or fructose (Figure 6A). It was determined that these substrates are not influencing the current response of the biosensor towards glucose. 

The effect of electroactive species (ascorbic and uric acids) was evaluated in a 10-times-diluted serum sample, adding a higher concentration than their normal physiological (0.141 mmol L^−1^ of AA [51,52] and 0.1 mmol L^−1^ of UA [53,54]). The diagram of registered current responses for the Ppy/GOx/DGNs/GR electrode in a 10-times-diluted sample of human serum after the addition of potentially interfering species is presented in Figure 6B. The obtained results with other real samples are presented in Table 2.

The increase of current responses is dependent on the type of real sample, interfering species, and their concentration. The addition of 0.01 or 0.05 mmol L^−1^ of AA increased current responses by up to 1.00 and 8.00%. The current responses obtained using the Ppy/GOx/DGNs/GR electrode after the addition of 0.01 or 0.1 mmol L^−1^ UA in human serum or saliva samples increased from 4.00 to 16.0% and from 5.00 to 13.0%, respectively, compared to the results obtained for 10.0 mmol L^−1^ of glucose without UA. The AA and UA at their physiological concentration did not significantly affect the current responses registered by the Ppy/GOx/DGNs/GR electrode in real samples. This indicates that the designed glucose biosensor, because of the formed Ppy layer, which was not permeable to negatively charged AA and UA anions, had an excellent anti-interference ability. The anti-interfering capacity of the biosensor presented here in human serum was higher than that described by our group for the biosensor based on the GOx/DGNs/GR electrode (the addition of 0.05 mmol L^−1^ of AA and 0.1 mmol L^−1^ of UA increased the current responses to glucose by 4.46 and 17.9%, respectively) [44] and similar for the biosensor based on the GOx/PANI-AuNPs _(6nm)_-GOx/GR electrode (0.01 mmol L^−1^ of AA increased the current responses to glucose by 2.11%) [48].

The applicability of a designed glucose biosensor based on the Ppy/GOx/DGNs/GR electrode was carried out in real samples. Usually, glucose concentration in the blood of a healthy person is in the range of 3.9 to 5.6 mmol L^−1^, and for diabetic patients, it can reach up to 30 mmol L^−1^ [3,54,55]. Glucose concentration in human saliva for diabetic patients is in the range of 20 to 200 μmol L^−1^ [14]. In our investigation, each real sample was measured four times by adding glucose, and the results were expressed as mean values (Table 2). Experiments were carried out to test the effects of the matrix, where the recovery ratio was in the range of 94.4 to 99.1% and the relative standard deviation was between 3.00 and 4.81%. Improved by the polymeric layer, the glucose biosensor could be successfully applied for glucose sensing in real samples.

The main advantages of the designed glucose biosensor based on the Ppy/GOx/DGNs/GR electrode are: (i) fast Ppy layer formation (22 h); (ii) appropriate sensitivity (59.4 μA mM^−1^ cm^−2^) and low value of LOD (0.070 mmol L^−1^); (iii) wide linear glucose determination range (up to 19.9 mmol L^−1^) and good repeatability (8.01%); (iv) good storage stability (*τ*_1/2_ = 33 days) and fast current response (5 s); (v) the suitability for the selective detection of glucose in real samples.

## 4. Conclusions

In this paper, we developed glucose biosensors based on a GR electrode modified by electrochemically synthesized DGNs and PANI or Ppy layers deposited by enzymatic polymerization. It was investigated that enzymatically formed Ppy was more suitable for the modification of the working electrode than PANI. Our investigations point out that newly designed glucose biosensor based on the Ppy/GOx/DGNs/GR electrode exhibits quick response, appropriate sensitivity, low limit of detection, rather wide linear glucose determination range, good reproducibility, and storage stability. This paper demonstrated a successful practical application of the designed biosensor for the determination of glucose concentrations in real samples containing some interfering species. The methodology presented in this paper can be used for the fabrication of glucose biosensors suitable for biomedical purposes and for application in food and beverage control as well as for the development of glucose-powered biofuel cells.

## Figures and Tables

**Table 1:** Comparison of gold derivative-based glucose biosensors.

Modified Working Electrode *	LOD (mmol L^−1^)/Sensitivity (μA mM^−1^ cm^−2^)	Linear Determination Range (mmol L^−1^)	Transduction System/Mediator	Reference
GOx/PDDA-rGO/MnO_2_/AuNPs/GC	0.0018/83.7	0.015–0.845	CV/K_4_[Fe(CN)_6_]	[9]
GOx/PANI/Au nanorods/Pt	0.0058/13.8	0.0176–1.00	CPA/–	[25]
GOx/DGNs/screen-printed	0.007/46.76	0.028–8.40	CV/K_4_[Fe(CN)_6_]	[19]
GOx/graphene/nano-Au/GC	0.017/56.93	0.2–2.00	Sweep voltammetry/–	[18]
GOx/rGO/β-lactoglobulin/DGNs/GC	0.0229/46.2	0.05–6.00	ChA/K_4_[Fe(CN)_6_]	[8]
GOx/HRP/carbon ink	0.03/–	0.05–1.00	ChA/K_4_[Fe(CN)_6_]	[12]
GOx/DGNs/GR	0.059/169	0.10–9.97	CPA/PMS	[44]
GOx/PANI-AuNPs_(6nm)_-GOx/GR	0.070/65.4	0.10–16.5	CPA/PMS	[48]
GOx/Ppy-AuNPs_(6nm)_-GOx/GR	0.071/55.4			
Ppy/GOx/AuNPs_(el depos.)_/GR	0.20/21.7	1.00–19.9	CPA/PMS	[23]
Chitosan-AuNPs nanocomposite/GC	0.370/–	0.40–10.7	CV/–	[13]
RBCM/PQQ/GDH/Au	1.06/–	2.50–10.0	ChA/–	[17] This work
Ppy/GOx/DGNs/GR	0.070/59.4	0.10–19.9	CPA/PMS	
PANI/GOx/DGNs/GR	0.18/43.9	0.30–19.9	CPA/PMS	

* GC—glassy carbon, GDH—quinoprotein glucose dehydrogenase, HRP—horseradish peroxidase, MnO_2_—manganese dioxide, PDDA—poly(diallyldimethylammonium chloride), PQQ—pyrroloquinoline, rGO—reduced graphene oxide, RBCM—red blood cell membrane.

**Table 2:** The main results obtained in real samples using a biosensor based on the Ppy/GOx/DGNs/GR electrode.

Real Samples	Current Responses * (%)				Glucose Concentration (mmol L^−1^)		Recovery Ratio (%)
	AA (mmol L^−1^)		UA (mmol L^−1^)		Added	Detected (*n* = 4)	
	0.01	0.05	0.01	0.1			
Human serum	102	102	104	116	2.42	2.38	98.3
					7.48	7.36	98.4
					11.0	10.9	99.1
Saliva	102	104	105	113	1.00	0.98	98.0
					9.92	9.78	98.6
Wine	100	103	–	–	2.99	2.85	95.3
					6.10	5.86	96.1
Coconut milk	104	106	–	–	4.48	4.30	96.0
Almond milk	102	106	–	–	4.48	4.37	97.5
					13.2	12.8	97.0
Apple juice	104	108	–	–	13.2	12.9	97.7
Mandarin juice	101	104	–	–	4.48 13.2	4.23 12.9	94.4 97.7

* Current responses were registered in real samples diluted with 0.05 mol L^−1^ SA buffer (pH 6.0), in the presence of 6 mmol L^−1^ PMS at +0.3 V vs. Ag/AgCl/KCl_(3 mol L_^−1^_)_; *n*—number of measurements.

## Data Availability

The data presented in this study are available on request from the corresponding author.

## Supplementary Materials

The following supporting information can be downloaded at: https://www.mdpi.com/article/10.3390/bios12080641/s1, Figure S1: Amperograms and voltammograms registered by the GOx/DGNs/GR electrode using CPA (**A**), SWV (**B**) and DPV (**C**) methods; Figure S2: The cyclic voltammograms registered by GR and GOx/DGNs/GR electrodes; Figure S3: The linear glucose determination ranges for four Ppy/GOx/DGNs/GR electrodes and their averages value after 22 h of polymerization; Figure S4: The linear determination ranges up to 1.0 mmol L^−1^ of glucose using bio-sensors based on the PANI/GOx/DGNs/GR and Ppy/GOx/DGNs/GR electrodes when polymeriza-tion was performed for 22 h.
