# Supplementary material for: Development and Practical Application of Glucose Biosensor Based on Dendritic Gold Nanostructures Modified by Conducting Polymers
Source: Biosensors (Basel). 2022 Aug 14;12(8):641. doi: 10.3390/bios12080641 (PMC9405657; doi:10.3390/bios12080641)
Supplement: Supplementary file 1 [file biosensors-12-00641-s001.zip › biosensors-1817774-supplementary.pdf]

biosensors

MDPI

Supplementary

Development and Practical Application of Glucose Biosensor Based on Dendritic Gold Nanostructures Modified by Conducting Polymers

Natalija German [1], Anton Popov [1,2], Arunas Ramanavicius [2,3] and Almira Ramanaviciene [1,2,*]

[1] Department of Immunology, State Research Institute Centre for Innovative Medicine, LT-08406 Vilnius, Lithuania
[2] NanoTechnas—Center of Nanotechnology and Materials Science, Institute of Chemistry, Faculty of Chemistry and Geosciences, Vilnius University, LT-03225 Vilnius, Lithuania
[3] Department of Physical Chemistry, Faculty of Chemistry and Geosciences, Vilnius University, LT-03225 Vilnius, Lithuania
* Correspondence: almira.ramanaviciene@chf.vu.lt

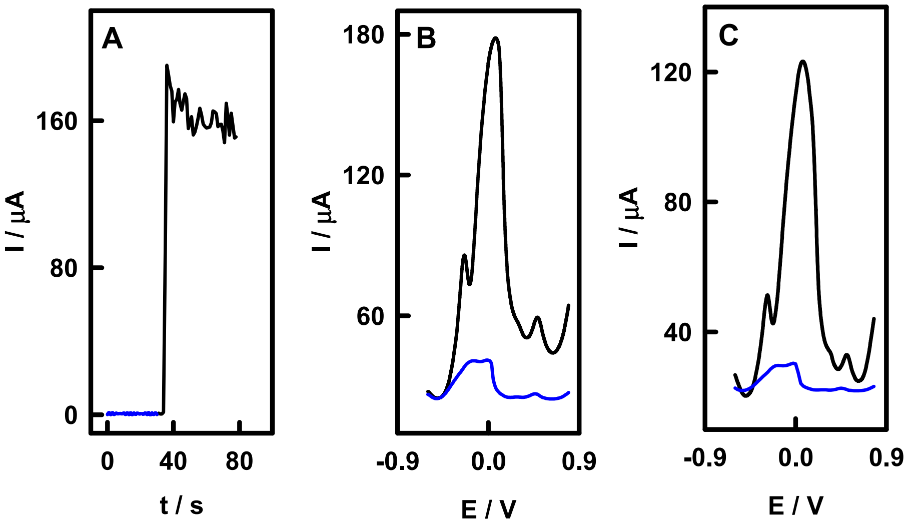

Figure S1. Amperograms and voltammograms registered by the GOx/DGNs/GR electrode using CPA (**A**), SWV (**B**) and DPV (**C**) methods. All measurements were performed in SA buffer containing 0.1 mol L^{-1} KCl, in the presence of 6.0 mmol L^{-1} PMS, without (blue line) and with (black line) 27 mmol L^{-1} of glucose.

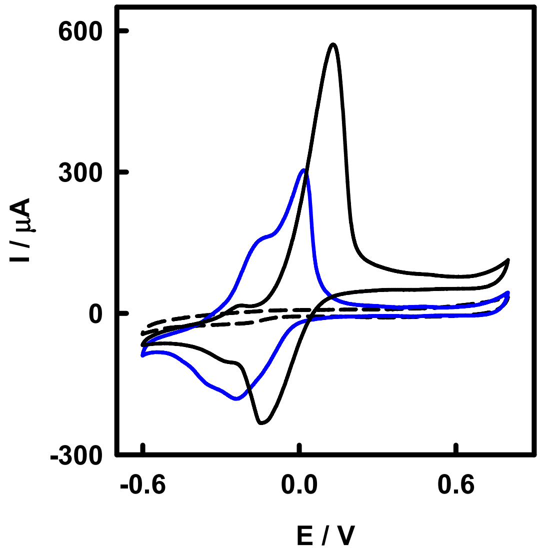

Figure S2. The cyclic voltammograms registered by GR and GOx/DGNs/GR electrodes. All measurements were performed in SA buffer containing 0.1 mol L^{-1} KCl, and bare GR electrode (dashed line) or GOx/DGNs/GR electrode in the presence of 6.0 mmol L^{-1} PMS without (blue line) and with (black line) 27 mmol L^{-1} of glucose.

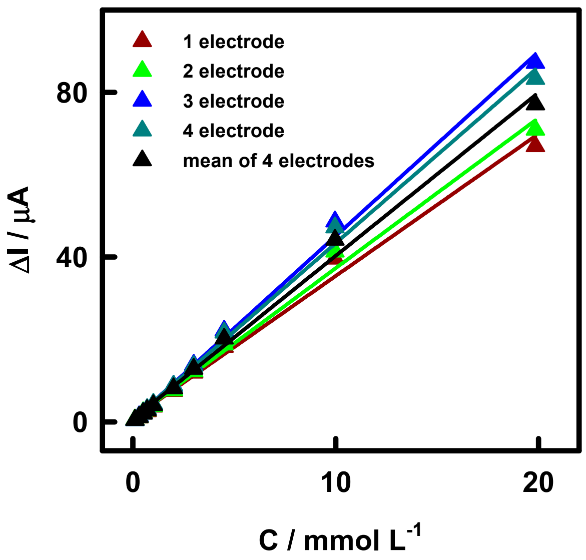

Figure S3. The linear glucose determination ranges for four Ppy/GOx/DGNs/GR electrodes and their averages value after 22 h of polymerization. All measurements were performed in SA buffer containing 0.1 mol L^{-1} KCl, in the presence of 6.0 mmol L^{-1} PMS.

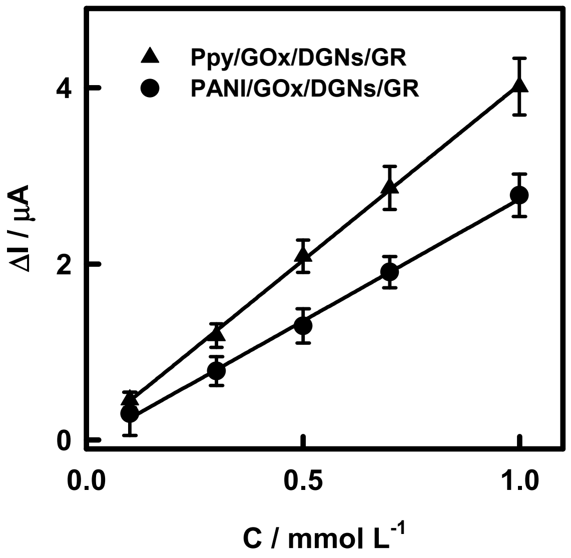

Figure S4. The linear determination ranges up to 1.0 mmol L^{-1} of glucose using biosensors based on the PANI/GOx/DGNs/GR and Ppy/GOx/DGNs/GR electrodes when polymerization was performed for 22 h. All measurements were performed in SA buffer containing 0.1 mol L^{-1} KCl, in the presence of 6.0 mmol L^{-1} PMS.
